# Acoustic Characterization of Transmitted and Received Acoustic Properties of Air-Coupled Ultrasonic Transducers Based on Matching Layer of Organosilicon Hollow Glass Microsphere

**DOI:** 10.3390/mi14112021

**Published:** 2023-10-30

**Authors:** Xinhu Xu, Liang Zhang, Hulin Guo, Xiaojie Wang, Lingcai Kong

**Affiliations:** 1 Thermometry Devision, National Institute of Metrology, Beijing 100029, China; xuxinhu@stumail.hbu.edu.cn; 2 School of Quality and Technical Supervision, Hebei University, Baoding 071000, China; wangxiaojie@hbu.edu.cn (X.W.); k18838711291@163.com (L.K.); 3 Zhengzhou Institute of Metrology, Zhengzhou 450001, China; guohulin@zim.ac.cn

**Keywords:** air-coupled transducer, composite, matching layer, flowmeter

## Abstract

An air-coupled transducer was developed in this study, utilizing hollow glass microsphere-organosilicon composites as an acoustically matching layer, which demonstrated outstanding acoustic performance. Firstly, a comparison and analysis of the properties and advantages of different substrates was carried out to determine the potential application value of organosilicon substrates. Immediately after, the effect of hollow glass microspheres with different particle sizes and mass fractions on the acoustic properties of the matching layer was analyzed. It also evaluated the mechanical properties of the matching layer before and after optimization. The findings indicate that the optimized composite material attained a characteristic acoustic impedance of 1.04 MRayl and an acoustic attenuation of 0.43 dB/mm, displaying exceptional acoustic performance. After encapsulating the ultrasonic transducer using a 3D-printed shell, we analyzed and compared its emission and reception characteristics to the commercial transducer and found that its emission acoustic pressure amplitude and reception voltage amplitude were 34% and 26% higher, respectively. Finally, the transducer was installed onto a homemade ultrasonic flow meter for practical application verification, resulting in an accuracy rate of 97.4%.

## 1. Introduction

Air-coupled ultrasonic transducers facilitate electric energy conversion into ultrasonic waves through air as the transmission medium. These transducers are widely used in intricate fluid measurement environments because of their non-contact measurement, high precision, and easy maintenance. They usually comprise piezoelectric ceramics, a matching layer, and a backing layer. When sound waves are emitted from the piezoelectric ceramic and enter the medium, the significant difference in acoustic impedance [1] causes most of the sound waves to reflect. To enhance transmittance and decrease the reflection of acoustic waves at the boundary, an acoustic matching layer is added between the piezoelectric ceramic and the air medium. Consequently, the ultrasonic transducers’ functionality depends significantly on the matching layer’s design.

In the realm of ultrasonic transducers, the optimization of acoustic transmission often starts from two aspects: the piezoelectric element and the matching layer. The usual materials for piezoelectric elements involve piezoelectric ceramics, piezoelectric polymers, and piezoelectric composites. Two types of piezoelectric ceramics exist: piezoelectric polycrystalline materials and piezoelectric single-crystal materials. The commonly used PZT [2,3,4] (lead zirconate titanate) serves as the piezoelectric polycrystalline material owing to its low raw material cost, high-piezoelectric constant, and mechanical durability. While piezoelectric single-crystal materials [5] are restricted to scientific research as they are costly to manufacture and possess limited usage, piezoelectric polymers have a wide range of applications, including PVDF films [6,7], which are well-known for their high mechanical strength and toughness. However, their issue with temperature instability still needs to be addressed. The composition of piezoelectric composites usually involves the combination of piezoelectric materials (typically PZT) and high polymers with different linkages, and there are various preparation methods available [8]. When optimizing matching layers, aerosol is an effective acoustic material due to its low single-phase density [9]; however, it is poorly malleable and challenging to work with. Kelly et al. [10] utilized a porous material composed of silicone rubber as a matching layer and compared its performance to not implementing a matching layer, resulting in a 30 dB signal improvement. Porous materials and aerosols have desirable acoustic properties, but their practical applications are limited by the plasticity and weak mechanical strength. Particularly within the field of ultrasonic flowmeters, which is the focus of this research, intricate gases in pipelines create a challenging environment. The matching layer, as the outermost layer, is in direct contact with the gases, imposing high requirements on its mechanical strength. Therefore, thermosetting polymers and lightweight, high-strength powder composites have received considerable attention for their exceptional acoustic properties and mechanical resilience. In research conducted by Park et al. [11], they employed an acoustic matching layer comprising of B-stage polymer resin and aluminum oxide powder which resulted in a 10% increase in the maximum sound intensity and a 37% increase in bandwidth for the transducer enhancement. Zhou et al. [12] utilized a hollow polymer-epoxy resin as the first matching layer and a polypropylene foam as the second matching layer, resulting in a 56% increase in the received amplitude when compared to commercially available ultrasonic transducers. Zhang et al. [13] showed the hydrogels’ remarkable acoustic wave transmission abilities, and then Kang et al. [14] used a matching layer consisting of a combination of hydrogels and hollow glass microspheres to achieve a characteristic acoustic impedance of 1.08 MRayl suitable for practical usage. Meanwhile, Fang et al. [15] utilized an anodized aluminum oxide template and an epoxy resin matching layer filled with type 1–3 to attain a 68% bandwidth at 6 dB. Gomez-Alvarez-Arenas et al. [16] used a ferroelectric electret thin film as a dual-mode active matching layer, which is innovative but still lacks practical confirmation. The organosilicon substrate that contains nanoscale metal powders has shown remarkable optical wave transmittance [17]. Its high strength and low density make it a potential substrate for ultrasound applications. Nevertheless, the possibility of using organosilicon as a matching layer for ultrasonic transducers has not been investigated. The objective of this study is to evaluate the viability of using organosilicon as a matching layer for ultrasonics. In addition, traditional methods do not differentiate between the transmitting and receiving capabilities of ultrasonic transducers during the test. To address this issue, our study conducts separate tests and analyses on both the transmitting and receiving performances of ultrasonic transducers to obtain a clearer understanding of their capabilities.

In this paper, the properties of three different substrates, epoxy resin, organosilicon and polyurethane, were first compared. Subsequently, we incorporated hollow glass microspheres of varying particle sizes, densities, and mass fractions into the substrates to analyze how they affect the acoustic properties of the matching layers. The ultrasonic transducer was enclosed within a PLA housing, and its transmit and receive performances were assessed and compared to commercially available transducers. The comparison revealed that the homemade ultrasonic transducer exhibited 34% and 26% higher transmit and receive amplitudes, respectively. Finally, we constructed a homemade ultrasonic flow meter using our homemade transducer to validate its practicality. Exceptional performance was demonstrated in our findings, showing significant potential for the advancement of air-coupled sensor technology.

## 2. Matching Layer Optimization Theory

In order to guide the design of the matching layer, it is first necessary to analyze the acoustic wave propagation theory.

Usually, in the analysis, the propagation of ultrasound is simplified to the propagation of a plane-travelling wave [18], and the following equation exists at any *x* position and time *t*:

(1) $$Z=\frac{U(x,t)}{V_{x}(x,t)}=\rho_{0}C_{0}$$

The constant *Z* in Equation (1) is derived from the ratio of sound pressure U(x,t) to normal velocity $\;V_{x}\left(x,t\right)$. This ratio is akin to the impedance in electricity and is commonly referred to as the acoustic impedance or specific acoustic impedance of the medium.

**Figure 1:**
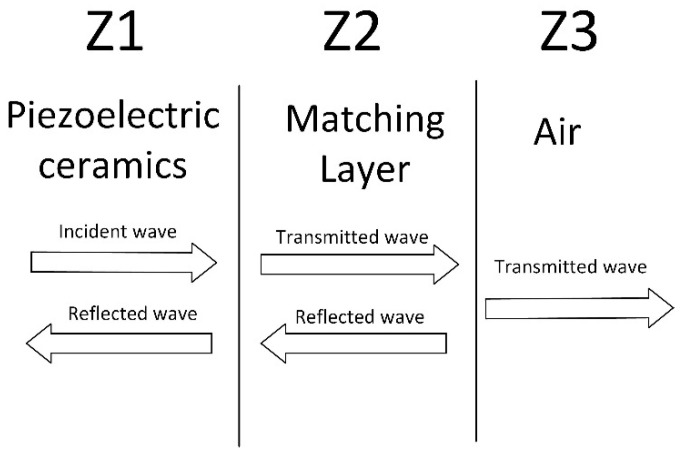
Transmission of ultrasonic transducer.

The theory of matching layer optimization is to introduce a matching layer medium 2 into the piezoelectric ceramic medium 1 and air medium 3, as shown in Figure 1, where the transmission coefficient can be expressed as

(2) $$T=\frac{4Z_{1}Z_{3}}{{\left(Z_{1}+Z_{3}\right)}^{2}{cos}^{2}k_{2}D+{\left(Z_{2}+\frac{Z_{1}Z_{3}}{Z_{2}}\right)}^{2}{sin}^{2}k_{2}D}$$

In Equation (2), $Z_{1},Z_{2},Z_{3}$ are the characteristic acoustic impedance of medium 1, medium 2, medium 3, respectively, $k_{2}=\frac{2\pi }{\lambda }$ is the wave number of medium 2, and *D* is the thickness of medium 2. When $k_{2}D=(n+\frac{\pi }{2})$, n is an integer, that means $D=\frac{\lambda }{4}$, the transmission coefficient is given by the following equation:

(3) $$T=\frac{4Z_{1}Z_{3}}{{\left(Z_{2}+\frac{Z_{1}Z_{3}}{Z_{2}}\right)}^{2}}$$

The derivation of Equation (3) with respect to $Z_{2}$ yields the maximum value of T taken when the conditions of the following equation are satisfied, the maximum value being 1:

(4) $${\;Z}_{2}=\sqrt{Z_{1}Z_{3}}$$

The maximum transmission coefficient is obtained when the characteristic acoustic impedance of the matching layer satisfies the conditions of Equation (4).

## 3. Fabrication and Testing of Ultrasonic Transducers

### 3.1. Selection of Piezoelectric Elements

The piezoelectric component is responsible for both generating and receiving ultrasonic waves in the ultrasonic transducer. Therefore, the selection of the appropriate piezoelectric material is crucial for the design of the transducer. In this study, commercially available and high-performance PZT piezoelectric ceramics were predominantly utilized.

PZT ceramics are available in various models with different compositional formulations, each with unique applications depending on their specific performance parameters. This study focuses specifically on the longitudinal telescopic vibration mode, the parameters of which are detailed in Table 1. PZT4 and PZT8 are characterized by their large longitudinal piezoelectric strain constant ${\;d}_{31}$, but relatively small longitudinal piezoelectric voltage constant $g_{31}$. As such, they are primarily utilized as emissive piezoelectric elements. The PZT5 series is commonly utilized as transceiver-integrated piezoelectric components due to its favorable longitudinal piezoelectric voltage constant $g_{31}$, making it suitable for this study. The piezoelectric ceramics utilized in this investigation were purchased from Baoding Hongsheng Acoustic Devices (Hongsheng Inc., Baoding, China) and measured ∅20*4 mm.

### 3.2. Matching Layer Sample Fabrication

#### 3.2.1. Substrate Material Preparation

Three substrates, epoxy, organosilicon, and polyurethane were used in this study and were mixed according to the manufacturer’s recommendations. The epoxy resin substrate was Epoxy-150W (Ausbond (China) Co., Ltd., Suzhou, China), with component A and component B mixed in a 5:1 mass ratio. The organosilicon substrate was CC1005 (Sirnice Inc., Dongguan, China), with component A and component B mixed in a 10:1 ratio. The polyurethane substrate was PU-130T (Ausbond (China) Co., Ltd., Suzhou, China), with component A and component B mixed in a 1:1 ratio. The mixing instrument was a ZYE-200VS (ZYE Inc., Shenzhen, China), planetary gravity mixer, and the rotational speed was set at 2000 rpm/min for 1 min. The substrate material samples were analyzed for their acoustic properties, with measurements of the solid sound velocity and density conducted using the echo method [19,20,21] and the drainage method, respectively. Additionally, the acoustic attenuation coefficient was measured with the ultrasonic transmission method, where the attenuation coefficient α is defined as

(5) $$\alpha =20\frac{lg\frac{A_{t}}{A_{r}}}{l}$$

where $A_{r}$ and $A_{t}$ are the initial incident and transmitted amplitudes, respectively, and l is the sample thickness. It is important to note that ultrasonic coupling agent is applied to both sides of the sample, while the sample is pressed firmly during the test to isolate the effect of air.

#### 3.2.2. Preparation and Testing Procedure of Hollow Glass Microsphere Based Matching Layer Samples

In this study, hollow glass microspheres were utilized as fillers in the preparation of composites. The objective was to examine the influence of varied mass fractions, densities, and particle sizes on the acoustic transmission properties of the acoustic matching layer. Four types of hollow glass microspheres, purchased from 3 M Company of the United States, with varying particle sizes, were used as fillers. The parameters used in the study are shown in Table 2. K20 and S15 have the same average particle size, but they differ in density (wall thickness). These differences were examined to determine the impact of density on the acoustic transmission properties of the acoustic matching layer. Additionally, K25 and K20 have the same density but differ in particle size, which was tested to explore the effect of particle size on the transmission performance of the acoustic matching layer. K1, which has the lowest density and largest particle size among the microspheres, is utilized to investigate the optimization effect of the glass microsphere on the acoustic matching layer under limiting conditions.

The prepared samples of the matching layer were tested for their density and solid sound velocity using ultrasonic echo and drainage methods. As a result, the values of acoustic impedance Z were obtained. The ultrasonic transmission method was used to test the acoustic attenuation coefficient of the matched coating samples.

### 3.3. Ultrasonic Transducer Assembly

**Figure 2:**
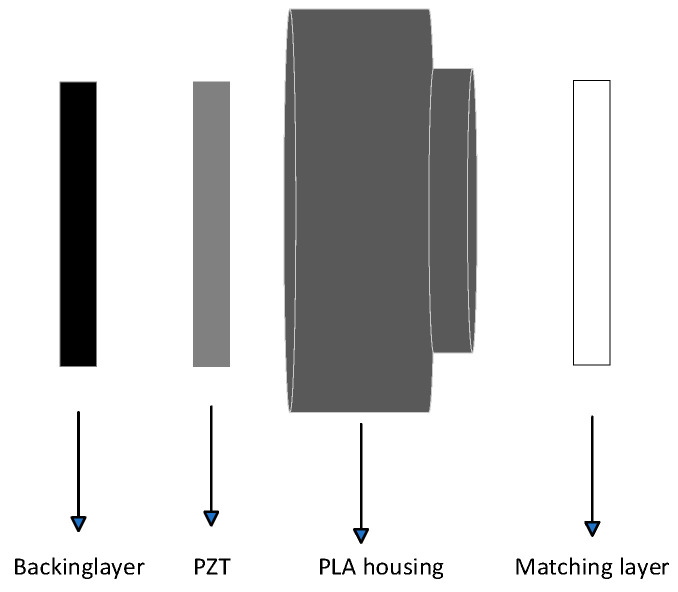
Ultrasonic transducer assembly.

Figure 2 displays the precise assembly procedure for the ultrasonic transducer, which consists of the matching layer, PLA housing, PZT piezoceramic, and the backing layer. A detachable mold of the desired thickness, coated with a release agent, is fabricated through a 3D printer. Then, the filler-mixed substrate is poured into the mold and solidified at room temperature. After the curing process was completed, we utilized a motorized sanding tool to sand the matched layer samples and measured their thickness with a micrometer to achieve the desired level of smoothness and thickness for the flattened surface. We used a 3D-printed PLA shell to create the housing. 

The typical function of the backing layer is to ensure unidirectional transmission of the ultrasonic transducer. In the domain of air-coupled transducers, an air backing layer is sufficient to meet the requirements. This is due to the significant acoustic impedance disparity between the piezoelectric ceramic and the air, leading to effective reflection of the ultrasonic waves emitted from the rear end. In this study, a black polyurethane CC1010 (Sirnice Inc., Dongguan, China) is utilized as a soft backing to encase the transducer, thereby maintaining its hermetically-sealed state, even in harsh external environments. Additionally, it guarantees unidirectional transmission at the same time.

### 3.4. Ultrasonic Transducer Emission Performance Testing

The mechanical damping of the transducer is often increased after the loading of the matching layer, the backing layer, and the shell, resulting in a series of mechanical behavioral changes, and this mechanical behavioral change may result in a decrease in the resonant frequency of the ultrasonic transducer, among other results. In this study, we conducted impedance tests on the fabricated ultrasonic transducers and assessed the changes in their mechanical behavior before and after optimization using the mechanical quality factor. The mechanical quality factor is defined as follows:

(6) $$Q_{m}=\frac{F_{s}}{∆F}$$

where $F_{s}$ is the resonant frequency and ∆F is the half-power bandwidth.

**Figure 3:**
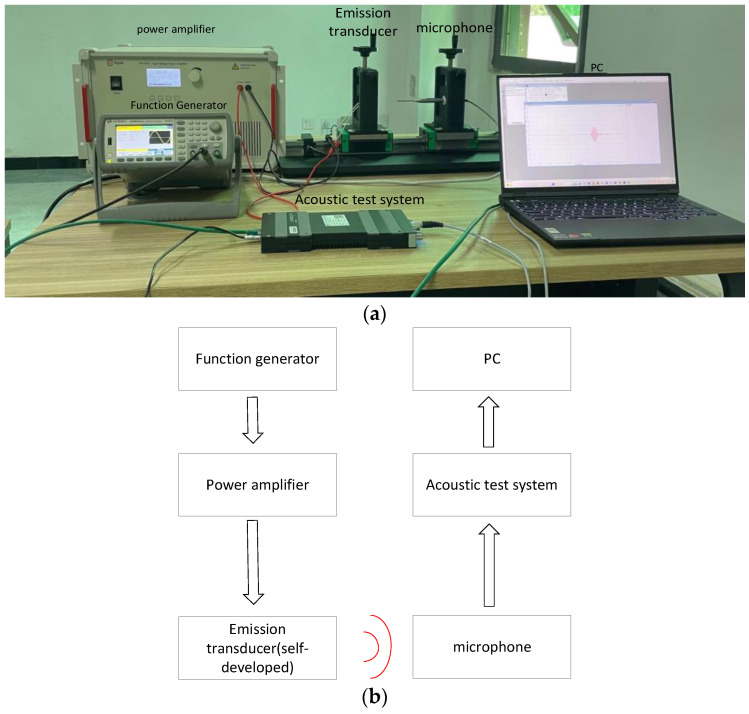
Device for measuring emission performance. (**a**) Experimental equipment table object; (**b**) Schematic diagram of the experimental equipment table.

As shown in Figure 3, the pulse signal from the function generator gets amplified by a power amplifier, which drives the ultrasonic transducer to emit ultrasonic waves. At the receiving end, the ultrasonic waves are received by a microphone type 4138-C-006 (Hottinger Brüel & Kjær Inc., Copenhagen, Denmark), and the signals are transmitted to a LAN-XI acoustic test system (Hottinger Brüel & Kjær Inc., Copenhagen, Denmark) for signal processing and presentation of acoustic signals (sound pressure) using Labshop software (Version 23.0.0.834-2020-01-10) on a computer.

To investigate the emission performance of the transducer under different excitation conditions, this study was carried out under different excitation voltages and different cycle counts of bursts, with the parameters shown in Table 3. To determine the electric–acoustic conversion efficiency of the ultrasonic transducer, a normalized coefficient, $K_{e}$, is defined as the equation below:

(7) $$K_{e}=\frac{P}{P_{i}}=\frac{IS}{\frac{U^{2}}{Z}}$$

where P and $P_{i}$ represent the acoustic and electric power, respectively, I is the acoustic intensity measured at a distance of 5 cm from the ultrasonic transducer, S is the radiation area, U is the excitation voltage, and Z is the impedance of the ultrasonic transducer.

Based on the results of the experiment, the best excitation conditions were chosen to measure the farthest transmission distance of the ultrasonic transducer. The farthest transmission distance was determined to be the point where the sound pressure was 10 pa. In conclusion, we compared the amplitude of the emitted sound pressure from the self-prepared ultrasonic transducer to the commercially available air-coupled ultrasonic transducer DYA-100-03H (Dayu Electronics Technology Inc., Fuzhou, China).

### 3.5. Ultrasonic Transducer Receiver Performance Testing

**Figure 4:**
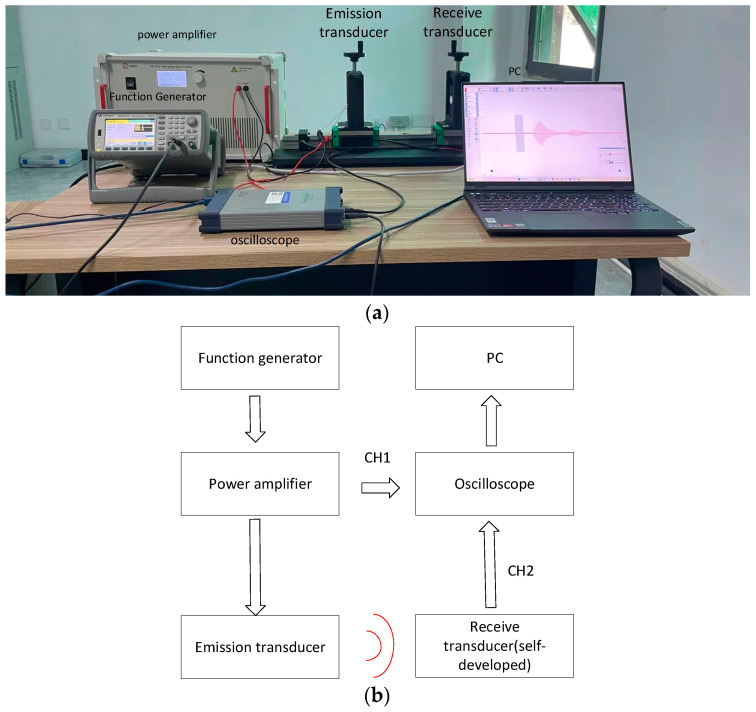
Device for measuring receiving performance. (**a**) Experimental equipment table object; (**b**) Schematic diagram of the experimental equipment table.

The receive performance test is illustrated in Figure 4. The same excitation method is applied as in the transmission performance test, with 200 Vpp and 10 burst cycles as the excitation signal. Additionally, the ultrasonic transducer which transmits ultrasonic signals is of the same type. The acoustic receiving performance of the ultrasonic transducer was evaluated at distances of 5 cm, 10 cm, 15 cm, and 20 cm using both homemade and commercially available transducers. The waveforms obtained from the oscilloscope were utilized to characterize the receiving performance.

### 3.6. Verification of Ultrasonic Transducers for Practical Applications

To confirm the practicality of the homemade ultrasonic transducer, it was installed in a differential time ultrasonic gas flowmeter [22,23] and tested using a Flowsick 600 series 8-channel ultrasonic flowmeter (Sick Inc., Waldkirch, Germany) as a standard reference flowmeter with a pipe diameter of 150 mm.

**Figure 5:**
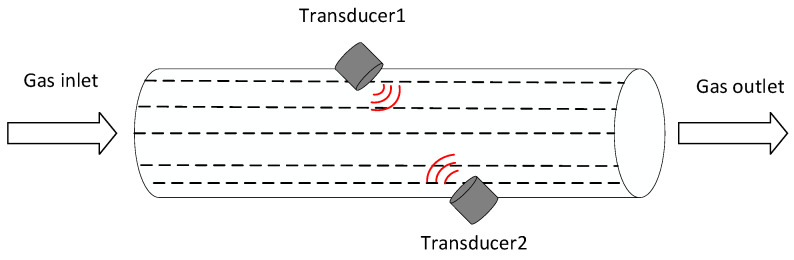
Differential Time Ultrasonic Flow Meter Schematic.

The time-difference ultrasonic flowmeter operates as shown in Figure 5 and calculates the fluid flow in the pipe by determining the difference between the downstream and upstream time, which is then multiplied by the pipe’s cross-sectional area to obtain the flow rate. Since the same type of homemade ultrasonic transducer is used for transmitting and receiving, the cis-countercurrent received waveforms show a very high correlation, and in this study, the velocity is obtained by calculating the time difference through the correlation [24,25,26] (Mutual Correlation Algorithm).

**Figure 6:**
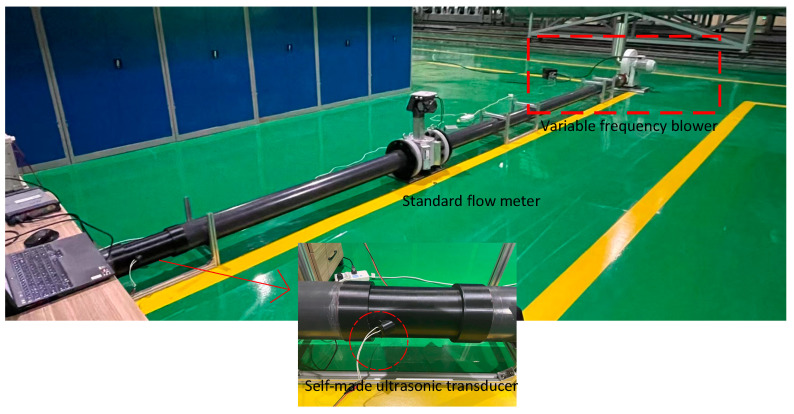
Ultrasonic transducer test system for practical applications.

Figure 6 depicts the system implemented for practical application testing. The system utilizes a variable frequency blower to provide adjustable wind velocity with a black PLA connection port that was fabricated using a 3D printer. To test the velocity and flow rate, a homemade ultrasonic transducer was mounted on the connection port. Finally, the results were compared to those obtained using a standard flow meter.

## 4. Results and Discussion

### 4.1. Result of Substrate Testing

**Figure 7:**
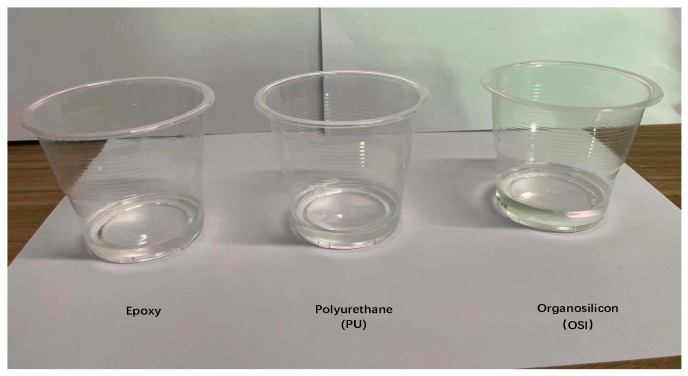
Curing effects of the three substrates.

Figure 7 displays the results of the three substrates after being cured, indicating that all three are transparent. Table 4 exhibits the results of the substrate examinations. Epoxy resins are commonly utilized as a substrate for the matching layers of ultrasonic transducers due to their cost-effectiveness [27], with a hardness up to Shore D70 or even higher. However, they have a higher acoustic impedance and acoustic attenuation. Compared to epoxy resin and polyurethane, organosilicon exhibits a lower acoustic attenuation coefficient and acoustic impedance, superior acoustic wave transmission performance, a Shore A70 hardness, and certain mechanical strength. These findings indicate that silicone substrates hold great potential for use in the production of the matching layers for ultrasonic applications.

### 4.2. Matching Layer Sample Test Results

**Figure 8:**
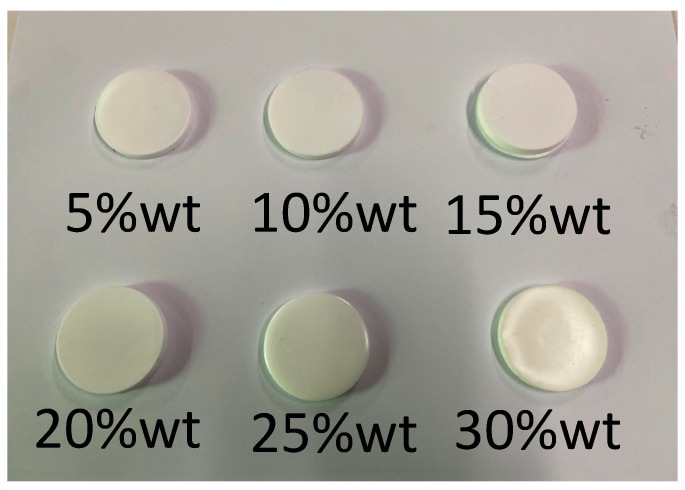
Matching layer samples of different mass fractions in kind.

**Figure 9:**
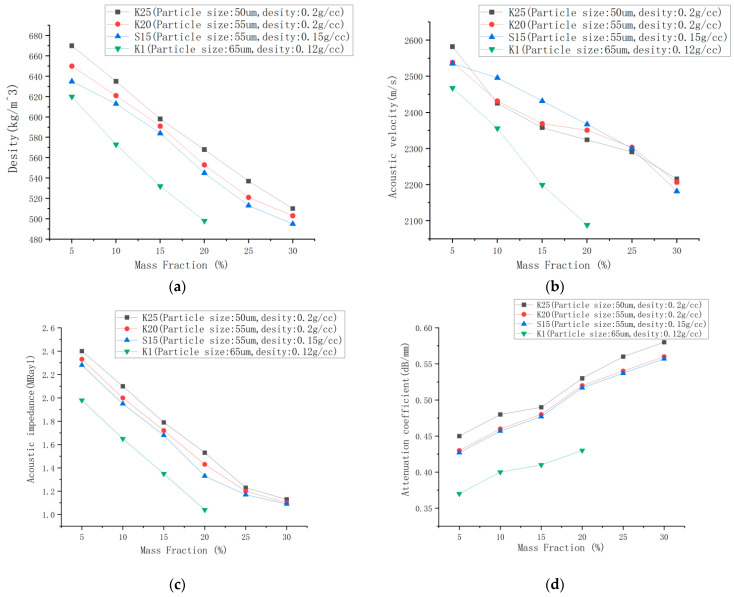
Matching layer acoustic test results: (**a**) Density of different samples; (**b**) Solid sound velocity of different samples; (**c**) Characteristic acoustic impedance of different samples; (**d**) Attenuation coefficients of different samples.

Figure 8 displays the matching layer samples with six different mass fractions, where the addition of hollow glass microsphere filler caused the substrate to change from a transparent color to pure white. The acoustic test results of four different hollow glass microspheres at mass fractions of 5%, 15%, 20%, 25%, and 30%, respectively, are shown in Figure 9. The K1 hollow glass microspheres were challenging to mix and were therefore not reported in this study for mass fractions exceeding 20%. From Figure 9a, it is evident that the density decreases with the increase of mass fraction. Additionally, comparing the K25 and K20 curves suggests that larger particle sizes have a more significant effect on reducing density. Similarly, comparing the K20 and S15 curves shows that fillers with lower density result in a more significant reduction in density. From Figure 9b, the graph of the sonic velocity in the solid sample shows that as the mass fraction increases, the sonic velocity decreases. However, within the 15–20% interval, there is a tendency for the slope to slow down. This can be attributed to the saturation of particle distribution within the filler of the solid. Figure 9c shows results from the sample’s acoustic impedance test, indicating a gradual decrease in acoustic impedance as the quality fraction increases, with the acoustic impedance reaching approximately 1 MRayl at the filling limit. Figure 9d displays the test results for the acoustic attenuation coefficient of the samples. It is evident that the attenuation coefficient increases with the increase in mass fraction. Additionally, upon comparison of different types of hollow glass microspheres, it can be observed that smaller particle sizes result in more significant scattering phenomena, leading to even more significant attenuation coefficients.

**Figure 10:**
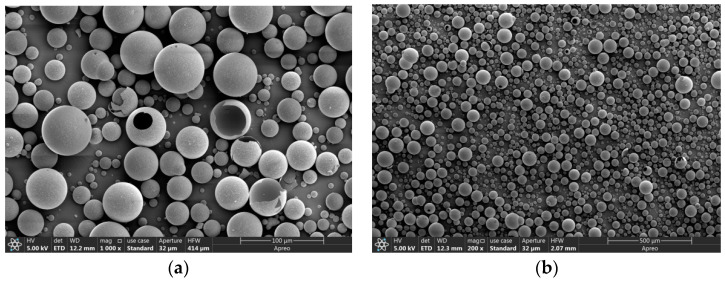
SEM image of samples: (**a**) 100 μm scale and (**b**) 500 μm scale.

Figure 10 shows the SEM images of K1 type hollow glass microspheres with a mass fraction of 20%. Figure 10a shows that some of the microspheres were broken due to extrusion by the centrifugal force generated during stirring, but most of them remained spherical. And Figure 10b shows that the distribution of the hollow glass microspheres was very homogeneous, which indicates that homogenization effect was good.

### 4.3. Transducer Emission Performance Test Results

**Figure 11:**
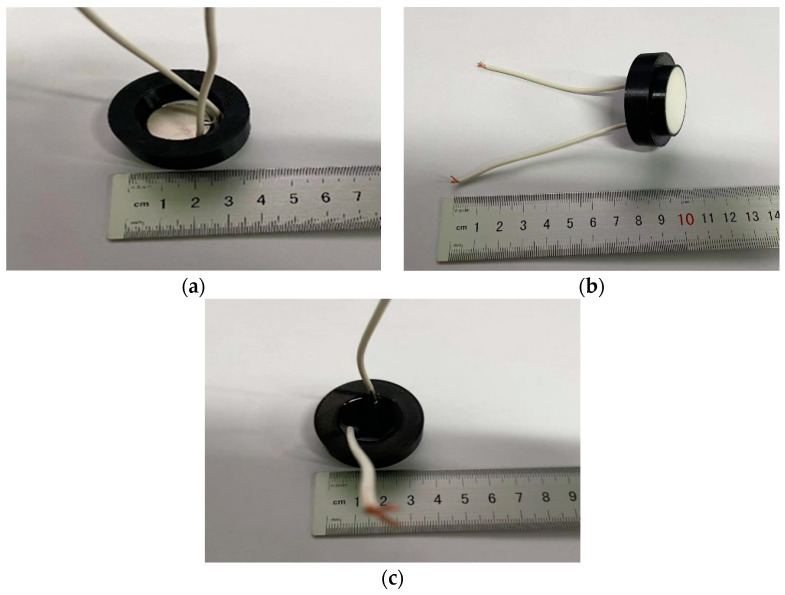
Transducer object. (**a**) Exterior of the transducer; (**b**) Interior of the transducer before encapsulation; (**c**) Object after Encapsulation.

As shown in Figure 11, the fabricated transducer was physically prepared with a black PLA package that encapsulated the piezoelectric ceramic with the matching layer, which was sealed with a black potting compound. Four ultrasonic transducers were created, each containing varying hollow glass microspheres with organosilicon matching layers, which were identified as K25-30, K20-30, S15-30, and K1-20, respectively, with mass fractions of 30% and 20%.

**Figure 12:**
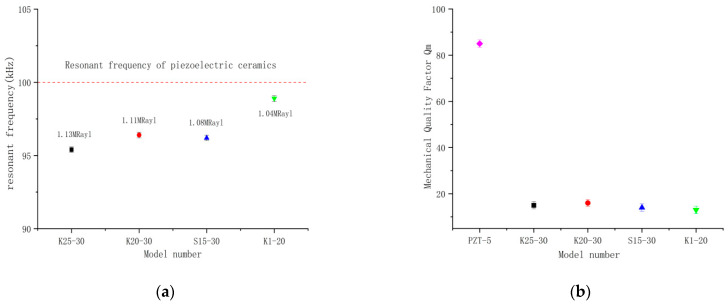
Changes in mechanical behavior before and after optimization. (**a**) Homemade transducer impedance test; (**b**) Mechanical quality factor test of Homemade Transducers.

A single piezoelectric ceramic possesses a resonant frequency of 100 kHz. Including a matching layer and an encapsulated housing, it results in a mechanical damping increase that induces a sequence of changes in mechanical behavior. Figure 12a illustrates that the resonant frequency of the four encapsulated models of ultrasonic transducer is slightly lower than that of the single piezoelectric ceramic due to increasing mechanical damping. As depicted in Figure 12b, the mechanical quality factor of all four models of ultrasonic transducers is significantly lower than that of the single piezoelectric ceramic. This reduction is attributed to the loss of energy transfer caused by mechanical damping, yet it also presents several advantages, including an expansion in the half-power bandwidth and a decrease in the tail-dragging condition, subsequently improving the precision of data processing at a later stage.

#### 4.3.1. Emission Performance Test Results for Different Excitation Voltages

**Figure 13:**
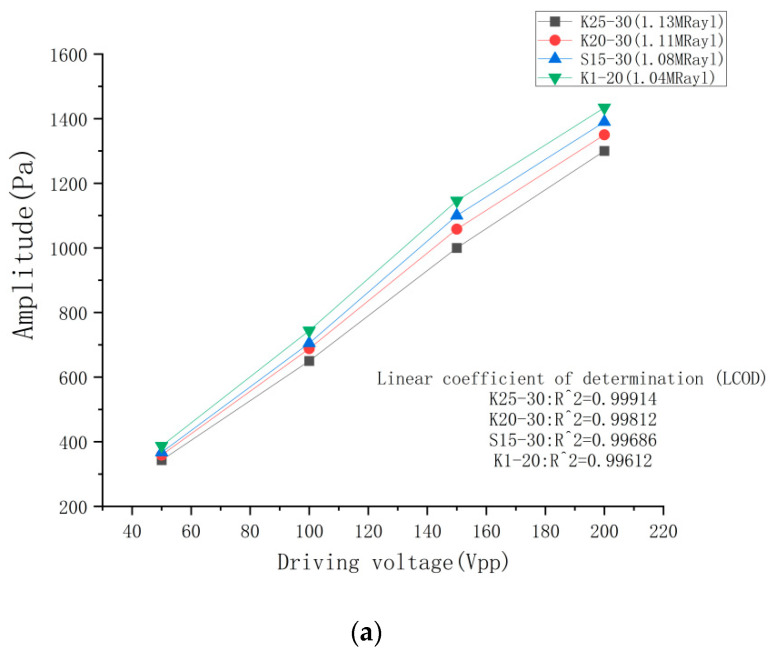

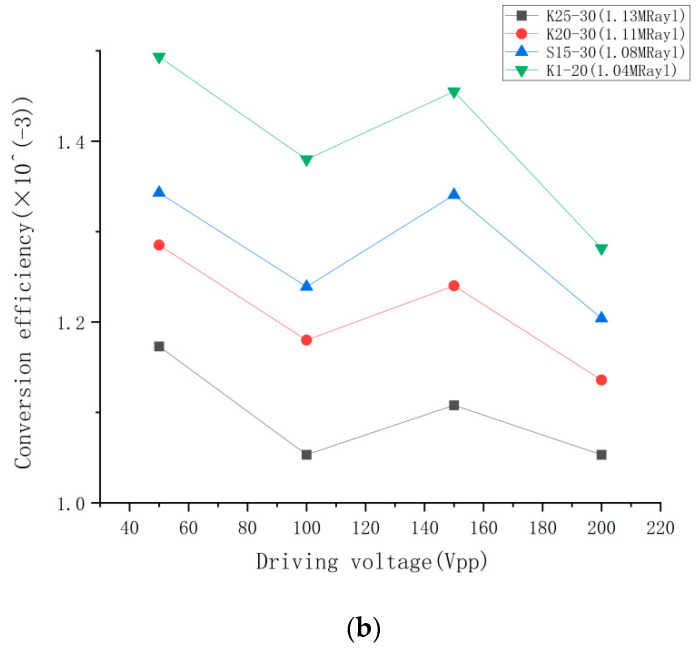
Test results for different excitation voltages. (**a**) Emission sound pressure results; (**b**) Electric-acoustic conversion efficiency results.

Figure 13 illustrates the test results of the four ultrasonic transducer models with four distinct excitation voltages. As shown in Figure 13a, the emission sound pressure of the ultrasonic transducer rises proportionally with the excitation voltage and displays a linear growth pattern, as indicated by the linear determination coefficient. K1-20 exhibits a better optimization effect compared to the other three ultrasonic transducer models. Figure 13b illustrates the electro–acoustic conversion efficiency results, demonstrating that at an excitation voltage of 50 Vpp, the electro–acoustic conversion efficiency is at its peak, but the resulting acoustic pressure amplitude is comparatively smaller. Meanwhile, at an excitation voltage of 150 Vpp, the electro–acoustic conversion efficiency remains high, and the sound pressure amplitude is adequate for more application scenarios. Thus, K1-20 boasts a higher conversion efficiency and sound pressure amplitude.

#### 4.3.2. Test Results of Emission Performance with Different Cycles of Burst

**Figure 14:**
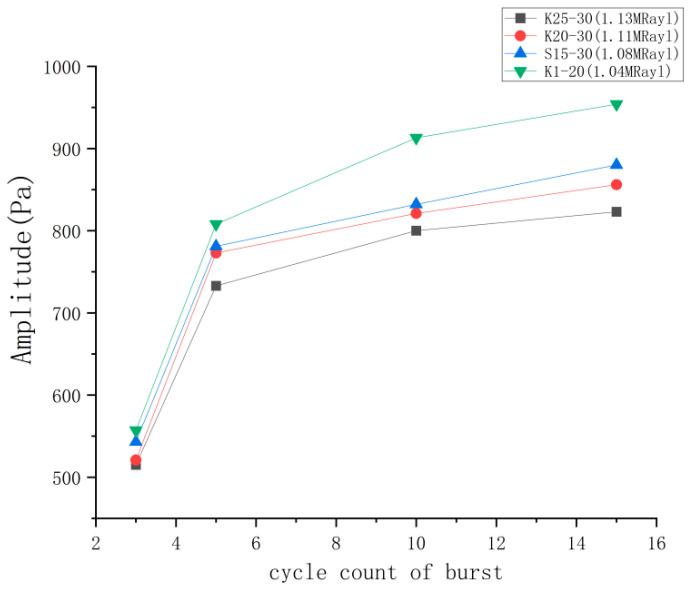
Emission sound pressure for different cycles of burst.

**Figure 15:**
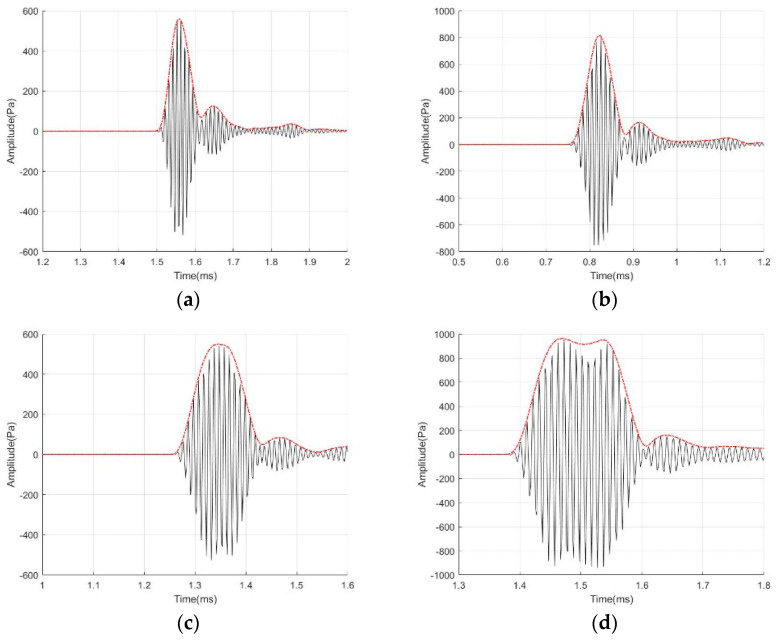
Emission sound pressure plots of K1-20 with different cycle count of burst: (**a**) cycle count of burst is 3; (**b**) cycle count of burst is 5; (**c**) cycle count of burst is 10; (**d**) cycle count of burst is 15.

Figure 14 displays four ultrasonic transducer models subjected to different burst excitation cycles. The plot indicates a direct relationship between the sound pressure amplitude and the count of cycles. However, the increase in amplitude from 10 to 15 pulses is minimal. Figure 15 displays the waveforms of the K20-20 model subjected to four diverse burst period excitation conditions. It is evident that an increase in the number of pulses results in a wider width of the waveform of the emitted sound pressure, rendering the peak unclear. A significant and smooth peak would result in a smoother envelope and allow the accuracy of the peak-finding operation in subsequent algorithmic applications. When the number of pulses reaches 10, the amplitude of the waveform increases, the peak value becomes more prominent, and the envelope curve appears smoother.

#### 4.3.3. Ultrasonic Transducer Maximum Emission Distance Results

**Figure 16:**
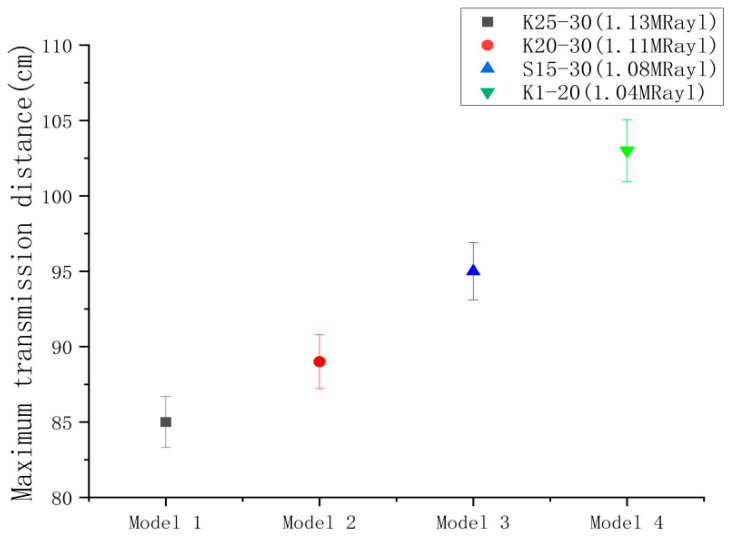
Maximum transmission distance results.

Based on the findings from the preceding sections, utilizing 150 Vpp and 10 cycles of burst as the optimal excitation conditions, the four models of ultrasonic transducers were stimulated to test the farthest transmission distance. The results, as shown in Figure 16, indicate that the ultrasonic transducer model K1-20 exhibits a longer transmission distance of 103 cm. As the transmission distance of the other models decreases, the reason for analyzing the cause is that there is a positive correlation between the farthest transmission distance and the acoustic pressure emission. Bigger acoustic pressure emission leads to a greater transmission distance.

#### 4.3.4. Comparison Test Results with Commercially Available transducer Emission Sound Pressure Amplitude

**Figure 17:**
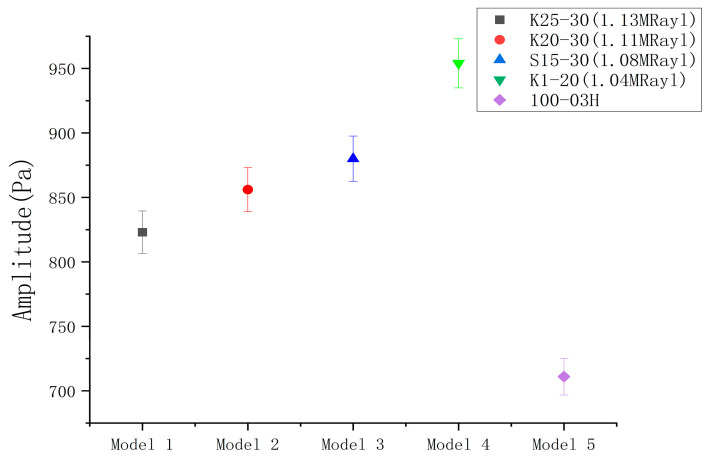
Comparison test results of emission sound pressure.

Figure 17 displays the emission sound pressure test results for four homemade ultrasonic transducers and commercially available transducers at a distance of 10 cm under 200 Vpp and 10 cycles of burst excitation conditions. The tests clearly demonstrate that the homemade transducers outperformed the commercially available transducers in terms of emission sensitivity. Additionally, the optimal K1-20 model exhibited a 34% increase in emission sound pressure amplitude compared to the commercially available 100-03H, which confirms the superior emission performance of the ultrasonic transducers fabricated in this study.

### 4.4. Transducer Receiving Performance Test

**Figure 18:**
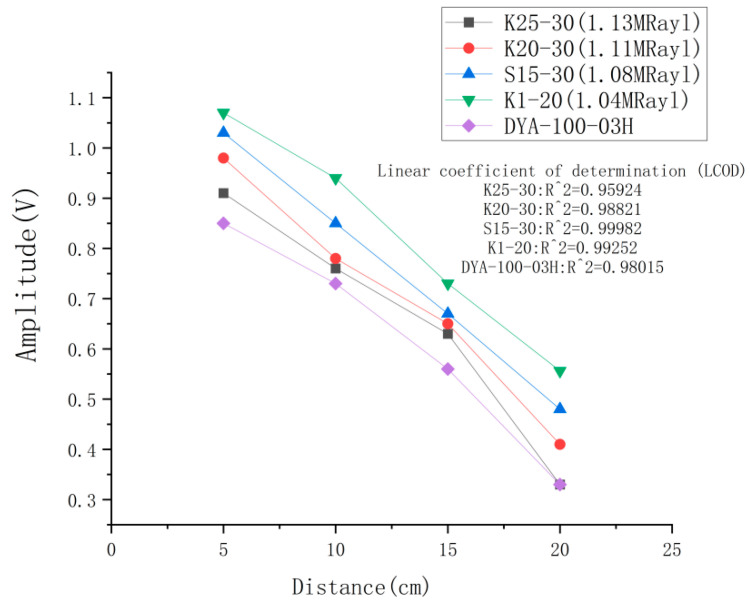
Receiving performance test results at different distances.

Figure 18 illustrates the received amplitude of a homemade ultrasonic transducer and a commercially available transducer at different distances. As the distance increases, the received amplitude of the ultrasonic transducer decreases almost linearly, as indicated by the linear coefficient of determination, and this attenuation is caused by the attenuation characteristics of air. According to the figure, the four homemade ultrasonic transducers outperform the commercially available ones in terms of receiving performance. Among them, the K1-20 model shows the highest receiving performance, which is improved by 26% compared to that of the commercially available transducers.

**Figure 19:**
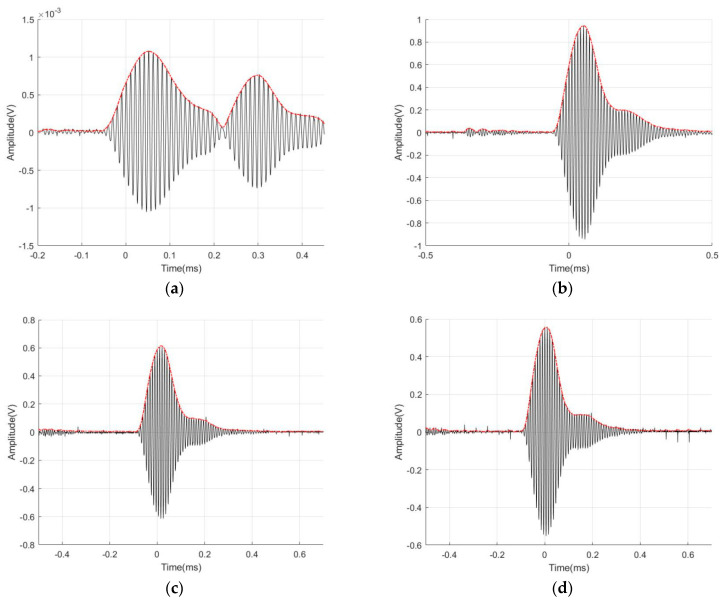
Received waveforms at different distances for model K1-20: (**a**) 5 cm; (**b**) 10 cm; (**c**) 15 cm; (**d**) 20 cm.

Figure 19 shows the K1-20 waveforms at different distances, with multiple waveforms not clearly separated at 5 cm. However, clear separation was achieved at 10 cm, serving as evidence that the homemade ultrasonic transducer is best suited for testing distances greater than 10 cm. Based on the findings in Section 4.3.3, K1-20 can be used within the range of 10 cm to 103 cm.

### 4.5. Practical Application Verification Test Results

**Figure 20:**
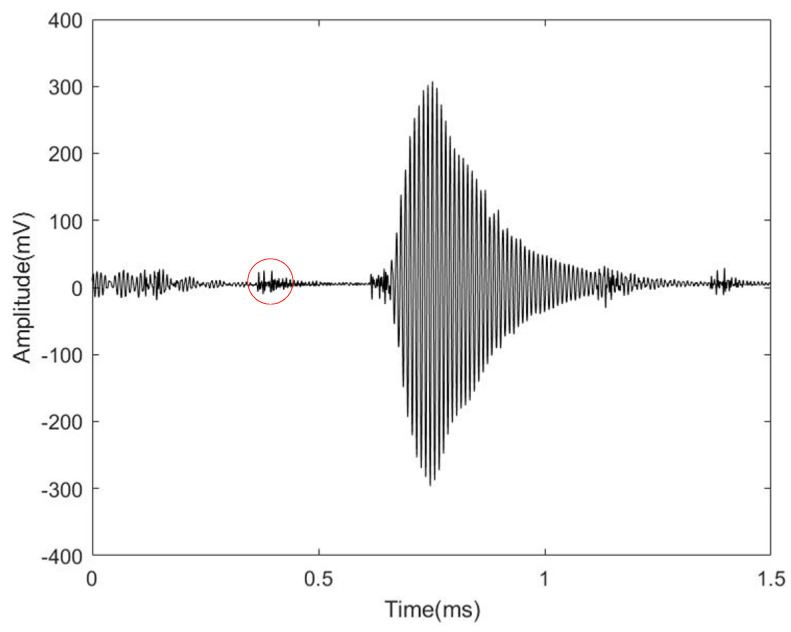
Waveforms of the receiver transducer of a homemade ultrasonic flowmeter.

**Figure 21:**
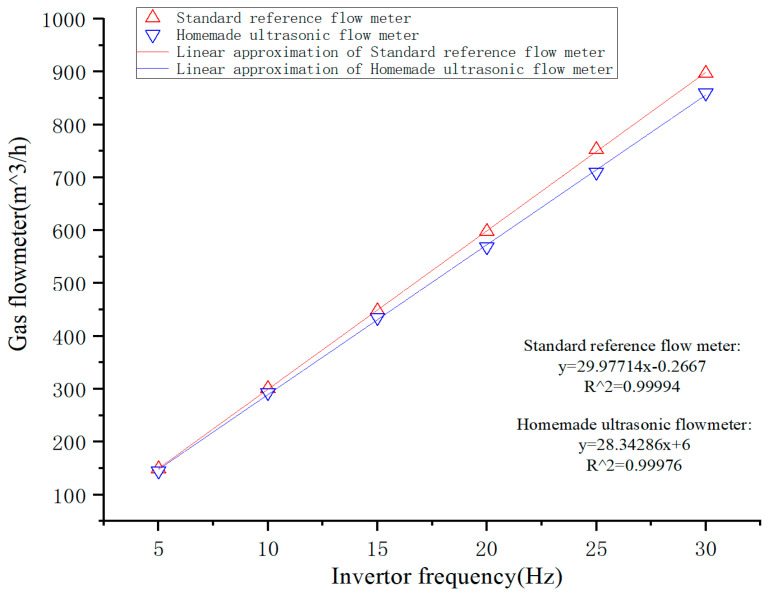
Gas Flow Measurement Results.

Figure 20 shows that when there is flow in the pipe, due to wind interference, the waveform at the receiving end will have some small burrs, but it can be seen that the amplitude of the small burrs for the actual received waveform is small, almost negligible, does not affect the judgment of the algorithm, this is all due to the gain in sensitivity. Figure 21 shows test results obtained through flow detection employing a homemade ultrasonic transducer. The flow rate elevates as the frequency of the frequency converter fan increases. The entire measurement area exhibits linear response characteristics. The decision coefficients of the standard reference flowmeter closely match those of the homemade ultrasonic flowmeter, with values of 0.99994 and 0.99976, respectively. Compared with the standard control flowmeter, the homemade ultrasonic flowmeter demonstrates an accuracy of 97.4% at flow rates under 600 $m^{3}/h$, and 95.8% at rates higher than 600 $m^{3}/h$. Verification through practical application reveals that the developed organosilicon hollow glass microsphere composite matching layer exhibits excellent air-coupling performance and holds promising prospects for future development.

## 5. Conclusions

In this study, after comparing the advantages and disadvantages of the three substrates, organosilicon was selected as the substrate for the matching layer of air-coupled ultrasonic transducer. The study examined the acoustic performance and physical properties of the matching layer in relation to diverse particle sizes and mass fractions. It was discovered that the optimum acoustic performance of the matching layer was achieved with an acoustic impedance of 1.04 MRayl and an acoustic attenuation of 0.43 dB/mm when the average particle size was 65 um and the mass fraction was 20%. It was also demonstrated that the mixture also has a certain degree of fluidity, which facilitated the fabrication process of the matching layer. For the fabricated ultrasonic transducer, the emission and reception performance tests were carried out, and compared with the commercially available transducer, the emission and reception performance were improved by 34% and 26%, respectively. At last, the practical application verification was carried out, which proved the good air-coupling performance and great application potential of the organosilicon hollow glass microsphere composites.

## Figures and Tables

**Table 1:** Piezoelectric Ceramics Parameters.

Name	*k_P_*	*d*_31_	*g*_31_	Curie Temperature (°C)	*Z_L_* (MRayl)
PZT-4	0.33	−120	−16	330	34
PZT-8	0.30	−100	−10	325	30
PZT-5	0.34	−175	−11	380	35

**Table 2:** Parameters of hollow glass microspheres.

Name	True Density (g/cc)	Particle Size Distribution			
		10th%	50th%	90th%	Average
K25	0.2	25	55	95	50
K20	0.2	30	65	110	55
S15	0.15	25	55	90	55
K1	0.12	30	65	110	65

**Table 3:** Emission Performance Test Parameters.

Test Paraments	1	2	3	4
Vpp (V)	50	100	150	200
Cycle count of burst	10			
Period of bursts (ms)	5			
Cycle count of burst	1	5	10	15
Period of bursts (ms)	5			
Vpp (V)	150			

**Table 4:** Comparison of different substrate parameters.

Material	Density $\mathrm{kg}/m^{3}$	Sound Velocity m/s	Acoustic Impedance MRayl	Hardness (Shore)	Attenuation Coefficient (dB/mm)
Epoxy	1250	3134	3.91	ShoreD 70	0.55
Polyurethane (PU)	1150	2980	3.42	ShoreA 50	0.48
Organosilicon (OSI)	950	3305	3.14	ShoreA70	0.25

## Data Availability

Data sharing not applicable.
